# The COVID-19 pandemic and organ donation and transplantation: ethical issues

**DOI:** 10.1186/s12910-021-00711-6

**Published:** 2021-10-21

**Authors:** Ban Ibrahim, Rosanne Dawson, Jennifer A. Chandler, Aviva Goldberg, David Hartell, Laura Hornby, Christy Simpson, Matthew-John Weiss, Lindsay C. Wilson, T. Murray Wilson, Marie-Chantal Fortin

**Affiliations:** 1 Canadian Blood Services, Ottawa, ON Canada; 2 Faculty of Law, University of Ottawa, Ottawa, ON Canada; 3 Canadian Donation and Transplantation Research Program, Edmonton, AB Canada; 4 Max Rady College of Medicine, University of Manitoba, Winnipeg, MB Canada; 5 Faculty of Medicine, Department of Bioethics, Dalhousie University, Halifax, NS Canada; 6 CHU de Québec—Université Laval Research Centre, Population Health and Optimal Health Practices Research Unit, Trauma-Emergency-Critical Care Medicine, Université Laval, Quebec, QC Canada; 7 Transplant Québec, Montreal, QC Canada; 8 Centre de Recherche du CHUM, Room R12-418, 900 rue St-Denis, Montreal, QC H2X 0A9 Canada; 9 Faculté de Médecine, Université de Montreal, Montreal, QC Canada

**Keywords:** COVID-19, Organ donation, Organ transplantation, Ethics

## Abstract

**Background:**

The COVID-19 pandemic has had a significant impact on the health system worldwide. The organ and tissue donation and transplantation (OTDT) system is no exception and has had to face ethical challenges related to the pandemic, such as risks of infection and resource allocation. In this setting, many Canadian transplant programs halted their activities during the first wave of the pandemic.

**Method:**

To inform future ethical guidelines related to the COVID-19 pandemic or other public health emergencies of international concern, we conducted a literature review to summarize the ethical issues.

**Results:**

This literature review identified three categories of ethical challenges. The first one describes the general ethical issues and challenges reported by OTDT organizations and transplantation programs, such as risks of COVID-19 transmission and infection to transplant recipients and healthcare professionals during the transplant process, risk of patient waitlist mortality or further resource strain where transplant procedures have been delayed or halted, and resource allocation. The second category describes ethical challenges related to informed consent in the context of uncertainty and virtual consent. Finally, the third category describes ethical issues related to organ allocation, such as social considerations in selecting transplant candidates.

**Conclusion:**

This literature review highlights the salient ethical issues related to OTDT during the current COVID-19 pandemic. As medical and scientific knowledge about COVID-19 increases, the uncertainties related to this disease will decrease and the associated ethical issues will continue to evolve.

## Background

The COVID-19 pandemic continues to have an impact on healthcare systems around the world. The organ and tissue donation and transplantation (OTDT) system is no exception and it faces multiple ethical challenges in its endeavours to make the best possible decisions with respect to recipients, donors, families, and healthcare teams [1, 2]. The speed of the pandemic’s onset, uncertainties such as the unknown risk of transmissibility from donors to recipients, the risk of infection to OTDT healthcare professionals, and the strain on hospital resources has led to rapid changes in the OTDT system, including the temporary closure of several living and deceased donor transplant programs [1, 3–5]. The ethical challenges associated with the COVID-19 pandemic also include the selection of recipients and donors outside of the normal allocation rules, issues relating to consent, legal issues, and resource allocation pressures [1]. Outstanding issues in the Canadian OTDT system relate to the use of COVID-19 infected donors—both the risk of transmission to recipients and the safety of the healthcare team retrieving those organs. To develop ethical guidance for our country’s OTDT systems and clinicians, a multi-disciplinary team was organized from within the Canadian OTDT community to focus on the ethical implications of COVID-19 [6].

Ethical principles that guide OTDT activity in Canada are based on an Ethics Consultation led by Canadian Blood Services in January 2011 [7]. Based on this consultation with key experts in OTDT, ethics, law, clinical practices and health policy, this report summarizes the key points of agreement. It lists foundational principles such as fairness, safety, accountability, collaboration, self-sufficiency, privacy, and cost-effectiveness. Table 1 summarizes these ethical principles and how they apply in the Canadian OTDT context. These principles are foundational and remain relevant for the functioning of the OTDT system, although they were not generated with the specific challenges of a global pandemic in mind. For this reason, we decided to undertake a review of the current ethics literature related to COVID-19 and OTDT.

**Table 1:** Ethical principles in OTDT based on CBS Ethics consultation [7]

Ethical principles	Definition
Self-sufficiency in organs	The obligation to increase the number of organ donors to meet the demand and prevent organ trafficking and transplant tourism
Population-based	Organs from deceased donors are a societal resource
Cost-effectiveness	Resources for the OTDT system should be used efficiently
Accountability	Stakeholders involved in OTDT are individually responsible for their actions
Collaboration and integration	Collaboration between all stakeholders to meet the healthcare needs of Canadians, and to integrate the opportunity for organ donation in all end-of-life care
Fairness	Canadian patients should be treated fairly, regardless of characteristics such as income, gender, location, etc. Efforts should be made to improve access to organ transplantation for disadvantaged populations
Security and safety	Since organs come from donors (living and deceased), there are inherent risks of disease transmission. Evidence and risk/benefit assessment should be considered in the decision whether to accept an organ
Privacy	Donor and transplant candidate information is confidential
Ethical practices in OTDT	OTDT should be aligned with Canadian values and should respect the altruistic nature of organ donation. OTDT should also be practised in an ethical manner that respects patients’ rights

The objective of this literature review is to summarize identified ethical issues and challenges associated with OTDT during the COVID-19 pandemic or other Public Health Emergency of International Concern (PHEIC) in relation to the three questions outlined below. We then compared this summary to the 2011 ethical principles, to identify potential gaps in those recommendations regarding OTDT systems during a PHEIC. The synthesis of these references has been used and will continue to inform ethical recommendations as part of the Canadian COVID-19 OTDT guideline development process and to provide guidance to clinicians facing challenges during this pandemic.

## Methods

Based on the stated objectives, and in consultation with a health information specialist, we designed a literature review of three overarching questions:

1. What ethical issues and challenges have been reported by organ donation organizations, living donation programs, and transplantation programs during the COVID-19 pandemic?
2. What impacts/challenges has the COVID-19 pandemic presented in relation to obtaining informed consent for organ and tissue donation and/or transplantation? Are there different approaches that should/can be considered?
3. What ethical issues have been identified for allocating organs and tissues during the COVID-19 pandemic?

The literature search was conducted with text and controlled vocabulary terms combining concepts for COVID-19/PHEIC, organ or tissue donation and transplantation, ethics, informed consent, and allocation. The following databases were used to retrieve articles and white papers: MEDLINE Ovid, Cochrane COVID-19 Study Register (https://covid-19.cochrane.org/), Embase Ovid, medRxiv and bioRXiv preprint servers Cold Spring Harbor Laboratory (https://www.medrxiv.org/; https://www.biorxiv.org/), Google Scholar/Google, NHS Blood and Transplant (UK) (https://www.nhsbt.nhs.uk/), organdonor.gov (US) (https://www.organdonor.gov/), European Commission (https://ec.europa.eu/health/blood_tissues_organs/organs_en), and Organ and tissue donation (Australia) (https://www.health.gov.au/health-topics/organ-and-tissue-donation). All study designs and published articles were included in the search strategy. Publications from January 1, 2002, to August 29, 2020 were included. We included publications from 2002 to capture publications that addressed challenges related to a previous coronavirus outbreak with the SARS-CoV-1 virus as it could assist with informing us on ethical challenges related to a public health emergency and organ donation and transplantation. Publications in languages other than English or French and publications that did not engage in a focused discussion of ethical issues related to OTDT were excluded. The initial search produced 891 articles. After removing duplications, 636 papers were screened by title and abstract. From these, 89 full texts were reviewed and 20 were included for data extraction. Table 2 provides details on articles included for analysis.

**Table 2:** Details of analyzed articles

Article name	Year	Topic (living donation vs. deceased donation, organ)	COVID-19 versus other pandemic	Location
Ethical issues in the COVID era: doing the right thing depends on location, resources, and disease burden [1]	2020	Living and deceased donation, kidney, pancreas/kidney, liver	COVID-19	Not applicable
Impact of the Covid-19 pandemic and lung transplantation program in France [2]	2020	Deceased donation, lung	COVID-19	France
Challenges in abdominal organ transplantation during the COVID-19 pandemic [8]	2020	Living and deceased donation, kidney, liver	COVID-19, SARS and MERS	China, United States
Practical considerations for solid organ transplantation during the COVID-19 global outbreak: the experience from Singapore [12]	2020	Living and deceased donation, lung, liver, heart	COVID-19, SARS	Singapore
Use of SARS-CoV-2-infected deceased organ donors: should we always "just say no?" [13]	2020	Deceased donation, liver, heart, pancreas, kidney, lung, Intestine, blood borne transmission, stomach, heart	COVID-19, SARS and MERS	China, Canada, United States, Italy
Benefit to few versus risk to many: an ethical dilemma during coronavirus disease 2019 pandemic for deceased-donor organ transplant in a resource-limited developing country [17]	2020	Living and deceased donation, kidney, liver, heart	COVID-19	India
Transplantation during the COVID-19 pandemic: nothing noble is accomplished without danger [20]	2020	Living and deceased donation, liver	COVID-19, SARS, Ebola	Italy
Utilization of deceased donors during a pandemic: argument against using SARS-CoV-2-positive donors [21]	2020	Deceased donation, blood transmission, liver, gastrointestinal, kidney, heart	COVID-19, SARS and MERS	United States
Briefing note: consensus guidance for organ donation and transplantation services during COVID-19 pandemic [22]	2020	Living and deceased donation, kidney, liver, lung, gastrointestinal	COVID-19	Canada, United Kingdom, Spain
Ethical considerations regarding heart and lung transplantation and mechanical circulatory support during the COVID-19 pandemic: an ISHLT COVID-19 task force statement [23]	2020	Organ donation, heart and lung	COVID-19	Not applicable
Liver transplantation in the time of COVID19: barriers and ethical considerations for management and next steps [24]	2020	Living and deceased donation, liver	COVID-19	United States, China, United Kingdom
NHSBT/BTS guidance for clinicians on consent for solid organ transplantation in adults, children and young people and living organ donation in the context of COVID-19 [25]	2020	Living and deceased donation	COVID-19	United Kingdom
High-immunological risk living donor renal transplant during the COVID-19 outbreak: uncertainties and ethical dilemmas [26]	2020	Living donation, renal	COVID-19	Singapore
Coronavirus disease 2019: utilizing an ethical framework for rationing absolutely scarce health-care resources in transplant allocation decisions [28]	2020	Organ donation, liver, heart, lung, kidney	COVID-19	United States
COVID-19 international recommendations for ODT [29]	2020	Living and deceased donation, donation programs	COVID-19	International
To procure or not to procure: hospitals face significant ethical dilemmas regarding organ donation during the COVID-19 pandemic [30]	2020	Organ donation	COVID-19	Not applicable
In service of the patient and not the virus: a pragmatic assessment of the approach to transplantation in South Africa during the COVID-19 pandemic [31]	2020	Organ donation generally	COVID-19	South Africa
Impact of the coronavirus (COVID-19) pandemic on Surgical practice—Part 2 (surgical prioritisation) [35]	2020	Organ donation generally, kidney, liver, heart,	COVID-19	United Kingdom
Transplant programs during COVID-19: Unintended consequences for health inequality [37]	2020	Organ transplantation	COVID-19	United States and United Kingdom
Needs must: living donor liver transplantation from an HIV-positive mother to her HIV-negative child in Johannesburg, South Africa [42]	2019	Living and deceased donation, liver	HIV	South Africa

## Results

Seven themes were identified and grouped under three categories: (1) general ethical issues and challenges reported by OTDT organizations during the COVID-19 pandemic; (2) ethical challenges related to informed consent for OTDT in the context of the COVID-19 pandemic; and (3) ethical issues related to organ allocation during the COVID-19 pandemic. The themes and categories will be described in more detail below. Table 3 summarizes the various ethical issues and associated ethical principles.

**Table 3:** Ethical issues related to COVID-19 pandemic and OTDT and CBS ethical principles

Ethical issues	Ethical principles
*General ethical issues and challenges reported by OTDT organizations and transplantation programs*	
Risk of COVID-19 transmission to transplant recipients	Safety and security, ethical practices in OTDT (non-maleficence and respect for persons)
Risk of COVID-19 exposure and infection to healthcare professionals during the transplant process	Safety, accountability, cost-effectiveness
Risk of waitlist mortality or further resource strain where transplants are halted	Safety, accountability, cost-effectiveness, ethical practices (non-maleficence)
Resource allocation	Safety, fairness, cost-effectiveness, ethical practices in OTDT (utility, non-maleficence, beneficence), population-based
*Ethical challenges related to informed consent for OTDT*	
Informed consent in the context of uncertainty	Safety, accountability, fairness, ethical practices in OTDT (respect for autonomy and beneficence)
Virtual consent	Fairness, ethical practices in OTDT (respect for autonomy), privacy, and accountability
*Ethical issues related to organ allocation*	Fairness, safety, population-based system, ethical practices in OTDT (justice, non-maleficence)

### Category 1: General ethical issues and challenges reported by OTDT organizations and transplantation programs.

Four themes were identified in this category: (1) risk of COVID-19 transmission to transplant recipients; (2) risk of COVID-19 exposure and infection to healthcare professionals during the transplant process; (3) risk of waitlist mortality or further resource strain where transplant procedures have been delayed or halted; and (4) resource allocation.

#### Risk of COVID-19 transmission to transplant recipients

The potential risk of transmission of COVID-19 from donors to recipients is frequently discussed in the literature. Although organ transplantation improves patients’ survival rate and quality of life, transplant professionals do not want to cause unnecessary harm by potentially exposing transplant recipients to a novel infection through the donor. There is still uncertainty about whether the use of an organ from a COVID-19 positive donor will result in transmission to the recipient, making safety a paramount concern in decision making [8]. Currently, there is a case report of donor to recipient COVID-19 transmission during lung transplantation [9]. However, the current risk of transmission is not calculable [10]. This risk is also minimized by the current recommendation to test all potential deceased donors for COVID-19 and to not proceed when donors have active COVID-19 infection [11].

Under the principles of non-maleficence, safety, and the precautionary principle, the possibility of infecting an immunosuppressed recipient (all transplant recipients receive intensive immunosuppression therapy post-transplantation) with COVID-19 through the transplant as well as the possibility of nosocomial COVID-19 infection during hospitalization might lead transplant teams to decide to delay or cancel transplant procedures [8, 12]. On the other hand, refusing organs from potential deceased donors who have active infection or have recovered from COVID-19 further limits an already small pool of potential medically eligible donors, thereby exacerbating the organ shortage.

A determination about whether to use organs from donors infected with COVID-19 is difficult. Arguments in favour include respecting the autonomy of candidates who desire to proceed with transplantation despite the potential risk of transmission [9, 13] balanced with honouring the decision of donors and families to donate. Conversely, potentially harming a recipient by proceeding with transplantation of an organ from a donor with an active COVID-19 infection goes against the wishes of donors and their families to help someone. Also, using organs from a COVID-19 infected donor requires consent from transplant candidates based on limited scientific understanding of the risk and may expose healthcare workers to a risk of COVID-19 infection [9, 13].

Although current recommendations, based on non-maleficence and safety principles, stipulate that organs from COVID-19 infected donors should not be used [11], there are case reports of liver transplantation from a COVID-19 infected donor and no infection in the recipient [14, 15]. There are also reports of ten kidneys transplanted from five donors with SARS-CoV-2 RNA positivity (but without any active disease) with no COVID-19 infection among the recipients and good outcomes [16]. Further research is needed to better document this risk and should be included in the information provided to future transplant candidates and donors’ families to make informed decisions. Now that COVID-19 vaccination is available, this risk should also be assessed in relation with the vaccination status of the recipient.

#### Risk of COVID-19 exposure and infection to healthcare professionals during the transplant process

An extension from the first theme, potential COVID-19 exposure for healthcare professionals is a common issue in the literature [8, 9, 13, 17]. This theme is not specific to transplantation and donation. A higher incidence of COVID-19 infection has been reported among healthcare workers compared to the general population [18, 19]. While a recipient may accept the risk of infection to gain the benefits of the transplant, it is also important to consider the impact of this decision on the health and safety of the medical team, particularly in situations where there is a shortage of protective equipment for the healthcare professionals [20], as was common during the early days of the COVID-19 pandemic and remains the case in some areas. Organ recovery from a COVID-19 positive donor also has the potential to expose numerous healthcare professionals both in and outside the operating room, and potentially contribute to community spread through their professional, personal, and community contacts [21]. Furthermore, when surgical teams need to travel to recover an organ, they might be exposed to a higher level of COVID transmissibility than in their home community, and if infected, potentially increase the rate of transmission when they return home [22]. In the event of transmission or potential exposure, donation and transplantation professionals will need to self-isolate; this puts a further strain on healthcare staffing and resources and affects other work that the professionals do (e.g., ICU staffing for donation, dialysis and post-transplant care for transplant teams) [17, 23]. It is therefore important to weigh the risks and benefits of using organs from a potentially COVID-19 infected donor from several perspectives.

#### Risk of waitlist mortality or further resource strain where transplant activity is halted

Halting or delaying transplant activity, even for a short time, increases the morbidity and mortality of waitlisted patients and could exacerbate the existing gap between supply and demand. Here, the survival of patients on the waitlist must be balanced against the safety of the community, both in and outside the hospital [8, 24]. Delays and interruptions also put a further strain on resources, as these patients may require access to other healthcare resources to maintain and control their current state of health [25].

Ho et al. [26] looked at considerations for performing renal transplants during COVID-19 and noted how both proceeding with a transplant or deferring it present risks of healthcare-associated infection (for example, in a dialysis centre for ESRD patients) or community-acquired infection. Halting kidney transplant programs could cause disappointments, increased vulnerability, and stress for patients and caregivers, as shown in a qualitative study with kidney patients in Australia [27]. As noted above, proceeding with transplant presents the risk of donor-derived infection, yet deferring can result in the risk of exclusion due to a patient’s disease progression or even death, or poorer post-transplant outcomes [26]. These considerations extend to other types of transplants as well [20].

#### Resource allocation

Prioritization and allocation of scarce resources is not a new issue for OTDT, as the demand for organs and tissues already exceeds supply, and because OTDT requires healthcare resources that are then not available to other fields of medicine. During COVID-19, the challenge of allocating healthcare resources and access to the ICU is much more prevalent [8, 28].

A lack of sufficient resources in the larger healthcare system may be a reason for temporarily suspending transplantation programs. Indeed, in certain exceptional circumstances, such as the COVID-19 pandemic, it may be reasonable to place a higher priority on allocating resources (material and human) for treating patients infected with COVID-19 rather than for transplant procedures and post-op care of newly transplanted patients. For example, during the pandemic, the need for intensive care unit (ICU) resources has substantially increased, with an impact on capacity to care for transplant patients who might also require these resources [23, 29]. However, it is important to consider that in some situations, transplanting a very sick transplant candidate needing an ICU bed may actually free up ICU resources, as the transplant could allow for recovery and transfer out of the ICU [29]. In an editorial from France, where most operating rooms were transformed into ICU rooms to accommodate COVID-19 patients in the spring of 2020, it was noted that using the remaining operating rooms “for 8-h long lung transplant procedures would constitute an unethical use of limited time and resources” [2]. This was considered unethical because using the remaining operating rooms would prevent their use for emergent surgeries and would use ICU resources, including professionals and technology, such as extra-corporeal oxygenation pumps that were urgently needed by COVID-19 patients [2].

Another issue related to resource allocation during the COVID-19 pandemic is whether it is morally acceptable to pursue deceased organ donation in a time of ICU resource scarcity. Maintaining a deceased donor requires mechanical ventilation, an ICU bed, and human resources. One could argue that these donors are competing with COVID-19 patients for ICU resources. On the other hand, taking care of a deceased donor will benefit and potentially save the lives of up to eight other transplant candidates. According to Potter et al. [30], this is a difficult ethical dilemma, but in certain circumstances it could be ethically acceptable for hospitals to use scarce resources to facilitate organ donations.

Maximizing benefit is repeatedly referred to in the literature as a guiding principle when dealing with scarce resource allocation during COVID-19. While this ethical principle exists in OTDT practice, during the pandemic, the principle is broadened (i.e., beyond OTDT programs) to include consideration of the pursuit of the greatest good for the greatest number of people, as opposed to the maximum benefit for one patient, with a shift towards a utilitarian concept of justice as the resource burden increases [31]. During non-pandemic times, OTDT programs strive to balance utility and fairness. Also, healthcare professionals have a duty to work in the best interest of their patients. During a pandemic, such as COVID-19, the interest of the population at large outweighs that of the individual patient [32]. This shift towards a more clearly utilitarian concept of justice has also been observed in the ICU setting with the development of triage protocols [33].

### Category 2: Ethical challenges related to informed consent for OTDT

Informed consent is of paramount importance in health care. The uncertainty and the novelty associated with COVID-19 makes the consideration of what informed consent means in this context particularly salient. The two themes in this category are (1) informed consent in the context of uncertainty and (2) virtual consent.

#### Informed consent in the context of uncertainty

The major issue highlighted for informed consent is the uncertainty surrounding COVID-19 transmission during transplantation and possible nosocomial infection following transplantation. To obtain informed consent, it is important to inform patients about procedures in place to rule out COVID-19 infection in donors and recipients as well as the potential risk of COVID-19 infection—to the extent that this is known—during transplant and post-transplant [28]. For instance, patients need to be informed that they as well as the donor will be tested for SARS-CoV-2 RNA. The current Canadian recommendation is to avoid transplantation from donors with active COVID-19 infection, particularly for lung transplantation. In the case of donors who were previously positive for COVID-19 and are currently asymptomatic, organ donation may be considered if the following conditions are met: (1) a minimum of 28 days after symptom resolution; (2) negative SARS-CoV-2 RNA tests from both the upper and lower respiratory tract within 48 h of donation; and (3) a review of the case by a transplant infectious disease physician [11, 34].

Patients also need to be informed about measures to mitigate these risks and the uncertainty related to the optimal management of COVID-19 infected recipients [1, 12, 22, 29]. In addition to informing patients about the risk of COVID transmission, they should also be informed about the risks of deferring the transplantation in terms of both morbidity and mortality. Other factors to be disclosed during the consent process are any limitations of and (possible) changes to the availability of post-transplant care, blood and blood products, follow-up appointments and re-admission pathways, requirement of and reasons for prolonged and strict isolation, and the importance of adhering to public health measures [24, 29, 35].

While there are many uncertainties about COVID-19 and the consent process, the basic principles of consent and the legal framework remain: an individualized risk–benefit discussion to confirm that a patient wants to remain active on the waitlist, the need to ask about and address patients’ concerns, confidentiality, giving a patient appropriate time to reflect on a decision, clear documentation of the consent discussion, and confirmation of consent in the patient’s record [25]. While consent should always be clearly documented, this becomes a more important requirement during the COVID-19 pandemic, considering the uncertainty related to this new virus [22].

#### Virtual consent

To minimize the risk of infection, consent for donation and transplant may be done virtually, through telemedicine for example, supplemented by physical and online documents. However, this way of obtaining consent raises new challenges, such as the possibility of miscommunication and misunderstanding (due to online format), digital illiteracy, and access to a stable internet connection for patients and families [25, 35]. It is also important to take into account that due to the evolving knowledge about the virus, documents for patient information may very quickly become obsolete, so it is important to provide patients with updates and to review their version of consent on admission to ensure the appropriate information is provided and to document the updated consent appropriately [25].

### Category 3: Ethical issues related to organ allocation during the COVID-19 pandemic

#### Social considerations in selecting transplant candidates

The question of who among the transplant candidates should get the organ is not a new problem in OTDT. All jurisdictions have developed allocation schemes to balance utility and fairness when deciding which patient should receive an organ from a deceased donor. The COVID-19 pandemic adds new challenges in the organ allocation process. One example is the requirement for recent transplant patients to physically distance and self-isolate after the transplantation (given the high level of immunosuppression after the transplant) in order to avoid complications associated with COVID-19 [36].This requirement could further disadvantage already disadvantaged patients in the transplant process, such as ethnic minorities or socio-economically disadvantaged patients [37], and be a source of mental distress [17]. Further, having a sufficient support system in place is also affected by the pandemic, as family support may be limited due to travel and gathering restrictions [17]. Technology such as videoconference and social media have been reported as a safe means to provide support while maintaining physical distancing but requires Internet access and digital literacy. As other research already demonstrates, the COVID-19 pandemic exacerbates existing health disparities, as the incidence of COVID-19 infection and mortality is increased among racial minorities and people of low socioeconomic status [38–40].

When transplant programs reopen after being interrupted during a pandemic, there could be a temptation to “cherry pick” transplant candidates, selecting those with the lowest risk of infection and who are able to comply with physical distancing and public health recommendations. This could prevent several categories of patients from having access to organ transplantation (older patients, patients with multiple co-morbidities, patients unable to comply with public health recommendations). This issue was not present in our literature review. Further research should be conducted to document this phenomenon, whether it occurs and what reasoning is used to both justify this approach and/or select patients in this manner, including possible ways to ethically mitigate such occurrences.

## Discussion

Since the beginning of OTDT, this field has dealt with ethical challenges. The COVID-19 pandemic has forced Canadian stakeholders in OTDT, among others, to re-examine the implications of upholding and balancing, to the extent possible, its foundational ethical principles of justice, autonomy, beneficence, safety, and accountability, among others. As in other areas of health care, the pandemic has led to renewed focus on some issues, such as the safety of staff and patients, which calls for heightened discussion of issues such as fidelity and reciprocity. The impact of the pandemic has not been uniform due to disparities in access to health and health care; these same disparities exist in OTDT and can be exacerbated by the pandemic.

The objective of this literature review was to summarize ethical issues and challenges associated with OTDT during the COVID-19 pandemic. As shown above, the principal issues are related to the risk of COVID-19 infection for the recipient and the healthcare providers, informed consent in an uncertain setting, resource allocation in a time of shortage of material and human resources, and the question of whether our organ allocation schemes should be modified, considering new requirements associated with the COVID-19 pandemic. At the beginning of the pandemic, some transplant programs stopped their activities, given the lack of resources and high rates of community transmission [41]. Although we are (at the time of writing) in the midst of a second and third wave of the COVID-19 pandemic, most transplant programs have been able to resume some or all of their transplant activities. As medical and scientific knowledge about COVID-19 increases, uncertainties related to this disease will decrease and the associated ethical issues will continue to evolve. Also, with the approval of new vaccines for COVID-19, issues of informed consent, the safety and effectiveness of vaccines for immunosuppressed patients, and the prioritization of individuals and groups for immunization will also be salient. Since our literature review was performed before the approval of these vaccines, we did not discuss vaccination in this review.

## Conclusion

The COVID-19 pandemic has also highlighted how we shift from beneficence and respect of autonomy principles to a framework based on safety, risk management, and a stronger utilitarian concept of justice. Finally, to develop an ethical framework acceptable for all citizens, OTDT organizations should be adaptable, transparent, and responsive to the public on issues affecting donors, recipients, healthcare providers, and the overall healthcare system.

## Data Availability

The datasets analysed during the current study are publicly available on the following databases: MEDLINE Ovid, Cochrane COVID-19 Study Register (https://covid-19.cochrane.org/), Embase Ovid, medRxiv & bioRXiv preprint servers Cold Spring Harbor Laboratory (https://www.medrxiv.org/; https://www.biorxiv.org/), Google Scholar/Google, NHS Blood and Transplant (UK) (https://www.nhsbt.nhs.uk/), organdonor.gov (US) (https://www.organdonor.gov/), European Commission (https://ec.europa.eu/health/blood_tissues_organs/organs_en), and Organ and tissue donation (Australia) (https://www.health.gov.au/health-topics/organ-and-tissue-donation).
